# Delayed sleep phase cases and controls

**DOI:** 10.1186/1740-3391-6-6

**Published:** 2008-04-29

**Authors:** Daniel F Kripke, Katharine M Rex, Sonia Ancoli-Israel, Caroline M Nievergelt, Walt Klimecki, John R Kelsoe

**Affiliations:** 1 Department of Psychiatry, University of California, San Diego, La Jolla, California 92093-0667, USA; 2 Scripps Clinic Sleep Center, 10666 North Torrey Pines Road, La Jolla, California 92037, USA; 3 Department of Psychiatry 116A, VA San Diego Health System, 3350 La Jolla Village Drive, San Diego, CA 92161, USA; 4 The Scripps Research Institute, 10550 North Torrey Pines Road, La Jolla, California 92037, USA; 5 Department of Pharmacology and Toxicology, College of Pharmacy, University of Arizona, PO Box 210207, Tucson, Arizona, 85721-0207, USA

## Abstract

**Background:**

Delayed sleep phase disorder (DSPD) is a condition in which patients have difficulty falling asleep before the early morning hours and commonly have trouble awakening before late morning or even early afternoon. Several studies have suggested that variations in habitual bedtime are 40–50% heritable.

**Methods:**

We recruited a case series of 205 participants, along with 221 controls (DSPD-C) with normal sleep, roughly matched for age, gender, and ancestry. A representative sample of San Diego adults recruited some years before was already available to confirm the control group. Both DSPD and DSPD-C provided blood or saliva samples for DNA and completed extensive questionnaires about sleep habits, sleep history, family history, sleep quality, morningness-eveningness traits, depression, mania, and seasonality of symptoms. The DSPD group wore wrist actigraphs for a median of 13.2 days. The representative sample collected previously had undergone actigraphic recordings, from which 48 hours of data were generally available.

**Results:**

The DSPD and DSPD-C samples showed almost no overlap on morningness-eveningness scores. DSPD cases went to bed and arose about 3 hours later than the DSPD-C and the representative sample. DSPD cases reported more difficulties with sleep, poorer sleep quality, and more depression, but there was no significant difference in a history of mania. DSPD cases reported more family history of late bedtimes, but female DSPD reported that their fathers' bedtimes were later than the fathers of male DSPD.

**Conclusion:**

These results indicate a DSPD phenotype is familial and associated with unipolar depression.

## Background

Some people are characteristically "larks" who retire and awaken early or "owls" who stay up late and awaken late. Larks also tend to be "morning" types who are energetic early in the morning, whereas owls are "evening" types whose energy may increase late in the evening. These traits have been described with morningness-eveningness scales (MES) such as the Horne-Östberg Scale (HO) [1]. Though mild variations in habitual bedtime are often easily accommodated, extreme forms of morningness or eveningness may produce social distress and disability.

When distressing, the severe forms of morningness and eveningness are termed Advanced Sleep Phase Disorder (ASPD, the larks) and Delayed Sleep Phase Disorder (DSPD, the owls) [2]. Patients with ASPD may retire at ~8 PM or earlier and likewise arise around ~4 AM or earlier. Such sleep times may resemble those of an agricultural society without electric light. Early bedtimes often cause only minimal social distress, so patients with ASPD rarely complain to physicians. Patients with DSPD may be unable to fall asleep early enough to rest adequately before it is time for school or work, or they may be quite unable to report to school or work on time. Consequently, DSPD can be a disabling and socially isolating condition, unless the patients are able to fit their habits into an accommodating social milieu. A Norwegian study estimated the prevalence of DSPD at 0.17% [3]. A Japanese study estimated 0.13% [4]. The prevalence of definite DSPD among adults ages 40–64 years was estimated in a representative actigraphic survey of San Diego as less than 1%, though either trouble falling asleep or trouble waking up was reported by 24.6% and both symptoms by 3.1% [5]. Late bedtime is also a common problem among adolescents. Adolescents with late bedtimes obtain less sleep and have more trouble with school, more disciplinary problems, and more depression [6,7], though it seems less than 1% meet formal criteria for DSPD [8]. Bedtimes are also commonly late among college students: for example, average bedtimes for samples at a Midwestern college ranged from 01:25 to 2:05 AM [9,10]. Some college populations in Europe may retire even later, with 6.9% of men and 2.8% of women reporting being extreme evening types [11]. It seems that social, developmental and environmental factors modify the prevalence of DSPD.

Several studies have found that DSPD may be familial [12]. Despite the social and developmental influences, analyses have most commonly estimated the heritability of bedtime at 40–50% [13-19], though in a Hutterite sample, the estimated heritability of bedtime was not significant [20], and in the Framingham study it was only 22% [21]. Evidently, genetic polymorphisms influence an important component of a person's habitual bedtimes. ASPD is associated in some families with incompletely-dominant single-nucleotide polymorphisms (SNPs) within PER2 and CSNK1D, which are genes which comprise parts of the body's circadian clock [22-24]. Some polymorphisms in the PER3 gene (also a circadian system gene) have been described as associated with DSPD and eveningness, though there have been inconsistencies among reports [25-28]. Less replicated reports have noted associations of DSPD, or eveningness, with other circadian system polymorphisms [29-37]. Associations of sleep timing with the T3111C polymorphism in the CLOCK gene have been reported inconsistently, possibly because all authors agree that these associations are weak, and this polymorphism is in strong linkage disequilibrium with numerous other polymorphisms throughout the CLOCK gene [29,38-41]. All reported associations of polymorphisms with DSPD need further replication. Moreover, it appears plausible that genetic susceptibility factors with important contributions to DSPD may not yet have been discovered.

To further explore the genetic polymorphisms associated with DSPD, we recruited self-selecting samples of DSPD cases and a control group roughly matched for ancestral origin, age, and gender. A representative sample of actigraphically-recorded San Diego adults was also available from previous research [5,42,43].

In this report, we present a description of our DSPD sample and analyze how cases differed from the control samples. Identification of genetic differences between the case and matched-control samples is currently under way, and will be reported elsewhere.

Delineation of a DSPD case sample encountered several difficulties. The landmark descriptions of delayed sleep phase disorder suggested very late bedtimes as diagnostic criteria, e.g., after 0200. However, some patients with earlier bedtimes were included in early case samples, because they made similar complaints of inability to fall asleep at the desired time and inability to arise at the desired waking time. It soon became evident that defining an explicit bedtime criterion for DSPD would be almost impossible because of age, social, and geographic variations in what may be considered a "normal" and socially-desirable bedtime [44].

Both the International Classification of Sleep Disorders and DSM-IV rejected an explicit bedtime criterion for DSPD, requiring instead a mismatch or misalignment between the desired sleep and wake-up times and the hours when a patient was able to sleep [2,45]. A misalignment definition accommodates the diversity of patient complaints without being confounded by a rigid bedtime criterion, but this criterion has the scientific limitation that it may be impractical to determine the extent of misalignment or to define a patient's "desired and socially acceptable time" for sleep [2]. By such contemporary definitions, the presence of delayed sleep rests entirely on the clinician's interpretation of the patient's complaint. We feared that mismatch criteria alone might not function adequately for our scientific goals.

As a compromise, we relied on the Horne-Östberg scale as the primary method of identifying "evening types" comparable to DSPD. This had the advantage of an explicit semi-numeric scale which incorporates subjective judgments about when a patient feels energetic or active, but the scale does not require distress or misalignment. The Horne-Östberg criteria were originally calibrated with a young-adult British sample, perhaps including many students [1]. We noted that applying the original Horne-Östberg criteria to mid-American adults, ASPD appeared rampant but extreme eveningness was more rare [38], which was inconsistent with clinical experience. Samples from France, India and New Zealand have likewise called for a modification of the original Horne-Östberg criteria [44,46,47]. Eventually, with a goal of collecting a sample primarily for genetic research which would emphasize the heritable characteristics, we adopted a hybrid classification approach relying on modified morningness-eveningness scale (MES) criteria, complaints suggesting misalignment of sleep propensity, and a past history suggesting that the delayed pattern was constitutional rather than acquired or environmental in origin or attributable to comorbidities.

## Methods

### DSPD sample recruitment

Recruitment was targeted to the Southern California region, particularly San Diego County, to enable the investigators to make home visits when necessary. Recruitment took place between June, 2004 and February, 2007. Recruitment of DSPD participants utilized contacts with Southern California sleep physicians, word of mouth, media contacts, newspaper advertising, late night radio advertising, UCSD minority outreach programs, paid context-based internet ads, and free internet advertising. A web site was constructed to facilitate contacts, provide potential volunteers with information, and allow potential volunteers to self-screen themselves versus the Horne-Östberg Scale: those with scores ≤ 30 were encouraged to volunteer for the full study.

The investigators recognized that a sample of homogeneous ancestry would help to prevent false-positive genetic findings due to sample stratification. On the other hand, inclusion of participants of diverse ancestry may produce more generalizable findings, may identify a larger number of associated genetic polymorphisms, and may satisfy institutional goals for fairness in medical research. Balancing these considerations, our target was to achieve a sample roughly representative of the San Diego County ancestral origins.

Only participants 25 years of age or older were accepted (with a few exceptions), to reduce the likelihood that participants might acquire late hours entirely through the social influences of youth culture or a college environment. Elderly participants were accepted if there was no suggestion of dementia or other illness which might distort circadian rhythms, and if there was a life-long history of delayed sleep.

Potential participants were provided written and verbal information about the study and signed written informed consent under the supervision of the UCSD Human Research Protection Program. Subjects referred or self-referred as potential DSPD cases completed a series of questionnaires, contributed a sample of blood or saliva for DNA, and underwent a 2-week wrist activity and illumination recording, using a wrist-activity recorder.

### Horne-Östberg morningness-eveningness questionnaire

An American idiom rephrasing was adopted for the Horne-Östberg Scale [1,48]. This is a 19-item form yielding scores from 16–30 (extreme eveningness) to above 70 (extreme morningness), as originally defined.

### BALM

The Basic Language Morningness scale or BALM [49] was administered as a simplified-language revision of the 13-item Composite Scale (CS) [50]. The CS was reported to have superior psychometric properties to the HO scale as well as greater brevity.

### PSQI

The Pittsburgh Sleep Quality Index (PSQI) measures subjective distress with sleep quality [51]. A total score above 5 suggests complaints of poor subjective sleep quality, but does not necessarily correspond to curtailments or interruptions of sleep measured polysomnographically.

### Health questionnaire

A general health questionnaire (available from the authors) was expanded from the version used in a previous representative survey [42] to included items about sleep satisfaction, past-week bedtimes and waking times, difficulties falling asleep too early or too late, lifetime health including depression, mania, and sleep disorders, medical treatments for sleep disorders or affective disorders, and family history. This questionnaire included items asking if participants went to bed earlier or later than most people as a child and also earlier or later than most people throughout adult life. Average adult bedtime was also recorded. Medications a participant used were recorded in broad categories.

### Mania history

Participants completed the Mood Disorders Questionnaire, a lifetime screening scale for mania with very high specificity [52]. Any occurrence of mania would indicate the presence of bipolar disorder.

### QIDS-SR

The Quick Inventory of Depression Symptomatology Self Report (QIDS-SR) is a self-rating scale for current major depression [53,54] with high correlation to the psychiatrist-administered Hamilton Depression rating scale.

### SPAQ

The Seasonal Pattern Assessement Questionnaire (SPAQ) includes a global seasonality scale, which reflects the seasonality of mood and may suggest the presence of a seasonal mood disorder e.g., winter depression, a condition sometimes associated with a delayed sleep phase [55].

### Actigraph

Each DSPD participant was provided an Actiwatch-L wrist actigraph to wear for 2 weeks (Mini Mitter Respironics, Bend, Oregon). This device recorded an integrated sum of wrist accelerations every 1 minute (using an arbitrary scale) and an average of the illumination of the wrist, using a phototransducer comparable to a photometer. Participants also completed a sleep log every 24-hours, estimating bed times, wake times, naps, and those times when the Actiwatch was removed from the wrist. The participant's activity patterns were then graphed, using the manufacturer's software. In addition, each 2-week recording was scored for sleep-wake minute by minute, using an algorithm validated for a different actigraph [56], modified for the Actiwatch settings, and then corrected by hand when the sleep log and behavioral cues, including the illumination data, suggested that the computer scoring was likely in error. This algorithm and hand scoring have not been validated with the Actiwatch. A 24-hour cosine was fit to each time series for sleep-wake, activity, and illumination, using a least-squares technique. The mesors (fitted means) of the cosines were utilized as the best magnitude estimates of daily sleep, activity, and illumination exposures, since this procedure adjusted for off-wrist intervals, incorporated data concerning wakes-after-sleep-onset within the major sleep period, and reflected sleep episodes outside the major sleep period. The acrophases (fitted peaks) of the cosines were the best estimates of circadian timing.

### Clinical evaluation

Once all data were assembled, the principal investigator (DFK) reviewed the record of each participant who had volunteered as a DSPD case and recorded the participant's DSPD classification as 1) absolutely certain, 2) fairly certain, 3) questionable, 4) unlikely, or 5) very doubtful. The main criterion for classification was the HO score, recognizing that the criterion for definite evening type of ≤ 30 was too strict for the San Diego population. Confirmatory classification criteria included the score on the BALM, reported prior-week and adult-life bedtimes and awakening times, the actigraphic recordings, and whether the participant reported going to sleep "somewhat later" or "much later" than most people their age, both as a child and as an adult. Whether the participant reported distress about falling asleep, reported related social or vocational problems, or had sought medical attention for a sleep problem was also considered. The consistency of the data supporting a classification of DSPD was evaluated, together with the presence of depression, other mental illnesses, or other sleep disorders which might confuse the classification. However, if depression or other disorders had their first incidence after the onset of delayed sleep and did not appear to be causing the delay, these disorders were not considered exclusionary. As many DSPD patients cannot consistently report to work by 8–9 AM, evening or night shift work was not considered exclusionary if the history indicated that the delay in sleep occurred before shift work was adopted, and the delay tended to persist when the participant was off work.

DSPD participants were reimbursed $100 for their time and effort, which included completing the questionnaires, providing a blood or saliva sample, wearing the Actiwatch for 2 weeks, completing sleep logs, and associated travel.

### DSPD-control recruitment

While the DSPD case series was being assembled, the investigators recruited DSPD-Controls (DSPD-C) who claimed to have healthy and normal sleep. Control volunteers were recruited by word-of-mouth, outreach at health fairs and community meetings, the internet, and by a campus poster seeking volunteers who "sleep like a baby." Control recruitment was targeted, so far as possible, to match the ancestry of the case series.

Each DSPD-C received an explanation of the study and signed written informed consent. DSPD-C completed all the questionnaires described above and contributed a sample of blood or saliva, but they were not asked to wear the actigraphs or to provide sleep logs. Since control participation usually required less than 2 hours, they were reimbursed $25.

The principal investigator (DFK) reviewed the record of each participant volunteering as control. Based on their questionnaires, control volunteers were retrospectively rated as 1) certain DSPD, 2) possible DSPD, 3) neither, 4) possible ASPD, or 5) certain ASPD. Only those in classes 3 or 4 were included in these DSPD-C analyses.

### Representative sample

As part of an earlier research project, from 1990–1994, our laboratory had recruited a representative population sample of San Diego adults ages 40–64, using structured random telephone dialing. A smaller representative sample of women ages 19–39 was recruited in parallel. More detail on the recruitment and characteristics of this sample has been reported in prior publications [5,42,43,57]. Representative sample participants had worn an actigraph of different design for 3 nights and 2 days. Representative participants had also completed a questionnaire which included similarly-worded questions about bedtimes, awakening times, and sleep disorders, but they did not receive any MES scales. The values of this sample as an additional control were its population representativeness and the availability of actigraphic sleep measures, whereas the disadvantages were numerous differences in the recruitment procedures and data gathered. Recruitment of the representative sample was completed over a decade earlier in a narrower geographic targeting. No DNA had been obtained from this earlier sample.

## Results

### Sample numbers and ages

The available samples included 205 DSPD volunteers, 221 DSPD-C controls, and 318 participants in the representative San Diego sample. The recruited DSPD volunteers included 10% who were retrospectively considered unlikely or very doubtful to have true DSPD, as shown in Table 1, and these 10% were excluded from certain analyses. Of the DSPD-C, 202 were considered neither delayed nor advanced. Ten DSPD-C controls that were judged possibly delayed were excluded from the analyses, but 9 judged possibly advanced were included. At least 3 of the DSPD cases displayed free-running sleep-wake rhythms (non-24-hour rhythms), and in several others, a masked free-running component was suspected from inspection of the 2-week actigraph. Because free-running subjects were excluded from comparisons assuming synchronized habits (e.g., bedtimes, acrophases), and because various data (especially actigraphy) were missing in certain cases, most comparisons involved somewhat fewer subjects than the total groups. The mean age of the DSPD participants was 38.8 (SD 12.4, range 22–78) years, and the mean age for DSPD-C was 40.8 years (SD 13.7, range 23–82) years (not significantly different). The mean age for the representative sample was 48.1 (SD 10.4, range 19–64) years, which was significantly different from DSPD and DSPD-C. The DSPD, DSPD-C, and representative samples were 65%, 64%, and 60% female, respectively (not significant).

**Table 1:** Classification of DSPD cases

	**Number**	**Percent**	**Cumulative Percent**
**1 Absolutely Certain**	72	35.1	35.1
**2 Fairly Certain**	69	33.7	68.8
**3 Questionable**	44	21.5	90.2
**4 Unlikely**	16	7.8	98.0
**5 Very Doubtful**	4	2.0	100.0
**TOTAL**	205	100.0	

### Horne-Östberg scores

**Figure 1:**
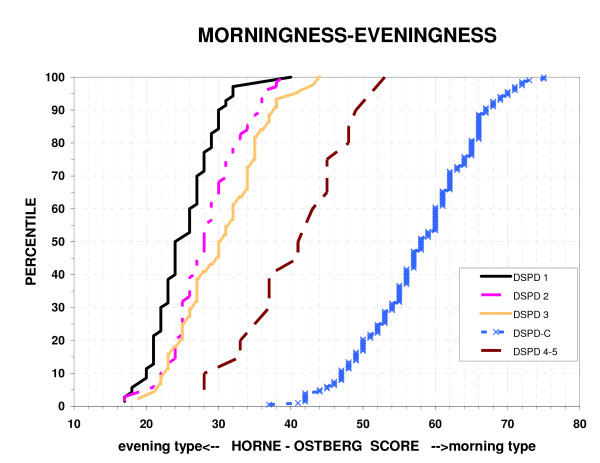
**Distribution of morningness-eveningness scores**. The percentiles of Horne-Östberg morningness-eveningness scores are plotted for 5 groups: a) absolutely certain DSPD 1, b) fairly certain DSPD 2, b) questionable DSPD 3, d) unlikely or very doubtful DSPD 4–5, and e) DSPD-C matched controls. Low HO scores indicate eveningness and high scores indicate greater morningness. In this study, the best criterion separating DSPD and DSPD-C was a score of 41.

As shown in Fig. 1, the HO scores of the DSPD volunteers rated certain, fairly certain, or questionable DSPD largely overlapped each other, but were very well separated from those of the DSPD-C group. Those DSPD volunteers rated unlikely or very doubtful to have delayed sleep phase disorder were intermediate, overlapping both the DSPD and DSPD-C groups. The Spearman Rank Order Correlation of reported age with the HO was r = 0.16 (P = 0.001), i.e., older participants reported slightly more morningness.

### Actigraphic results

From DSPD participants, 193 actigraphic recordings were available, ranging from roughly 5 to 14 days in duration. The median actigraphic recording was 13.2 days in duration, with the 10^th ^percentile 10.4 days and 90^th ^percentile 13.8 days. For the representative sample, there were 311 actigraphic data sets available. Although most of those original representative recordings included 3 nights and 2 days of data, to avoid biasing circadian data by time of day, 89% of these recordings were truncated to approximately 48 hours, but the rest were even shorter.

**Figure 2:**
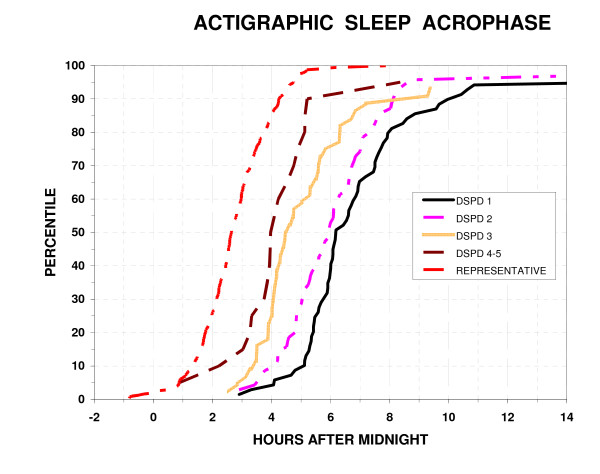
**Distribution of actigraphic sleep acrophases**. The percentiles with an actigraphic sleep acrophase at or below each time are plotted versus clock hours after midnight. The percentiles are plotted for 5 groups: a) absolutely certain DSPD 1, b) fairly certain DSPD 2, c) questionable DSPD 3, d) unlikely or very doubtful DSPD 4–5, and e) a representative population sample of San Diego adults.

Objectively, by actigraphic scoring, DSPD slept 417 ± SD 78 min. per 24 hr., while the representative sample slept 353 ± SD 71 min. per bedtime (P < 0.001, N = 194 and N = 301, respectively). Actigraphic sleep times did not differ by the certainty of the DSPD classification (classes 1 to 5). The DSPD data included daytime sleep and "naps", but the representative sample had little sleep out of bed, << 15 min. in most cases, so this had not been scored. The mean sleep acrophase (approximating the mid-sleep time) was 5:56 AM (SD 2.3 hours) for DSPD and 2:44 AM (SD 1.2 hours) for the representative sample (P < 0.001). Thus, the DSPD group's sleep acrophase was more than 2 standard deviations later than the representative sample and much more variable: Levene's Test rejected equality of variances (P = 0.001.) As illustrated in Fig. 2, subjects classified with DSPD with greater certainty had later sleep acrophases, but even the groups rated 4 or 5 (probably or definitely not DSPD) had substantially later sleep acrophases than most of the representative sample.

Likewise, wrist activity acrophases were 16:55 (SD 2.1 hours) for DSPD and 13:47 (SD 1.4 hours) for the representative sample (P < 0.001), Levene's test for equality of variance P < 0.001. Similarly, the light acrophase was 15:07 (SD 1.9 hours) for DSPD and 12:59 (SD 1.5 hours) for the representative sample (P < 0.001), Levene's test for equality of variances not significant (NS). Note that scored sleep and wrist activity displayed about the same degree of delay among DSPD participants compared to the representative sample, but their wrist light exposures were not correspondingly as late. The mesor fitted to log_10 _[lux] for the DSPD participants was 0.93, whereas that for the representative sample was 1.01 (P = 0.001, two-tailed t test), but the representative sample had a greater range (Levene's Test for Equality of Variances P = 0.001). This difference corresponded to about 20% greater illumination for the representative sample, about 4% of the total range of illumination exposures in the samples, the difference in samples representing an effect size of 0.31.

### Reported bedtimes and uptimes

**Figure 3:**
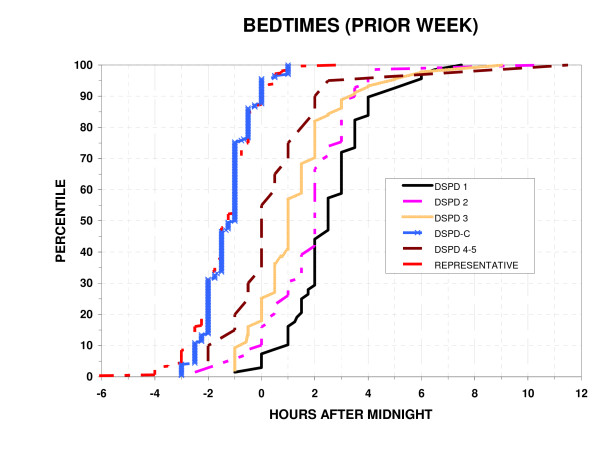
**Distribution of bedtimes**. The percentiles with a reported bedtime (week prior to completing the questionnaire) at or below each time are plotted versus clock hours after midnight. The percentiles are plotted for 6 groups: a) absolutely certain DSPD 1, b) fairly certain DSPD 2, c) questionable DSPD 3, d) unlikely or very doubtful DSPD 4–5, e) the DSPD-C, and f) a representative sample of San Diego adults.

**Figure 4:**
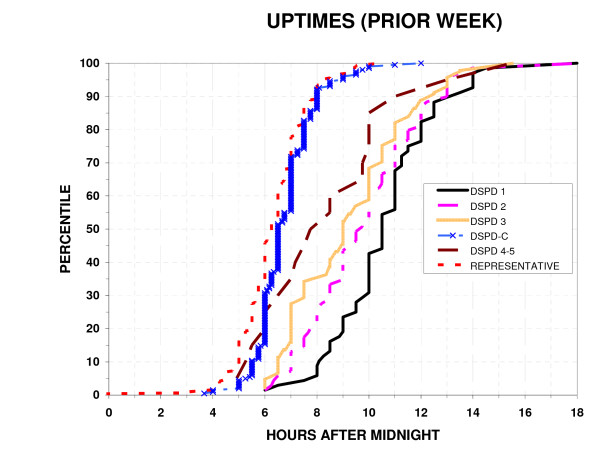
**Distribution of wake-up times**. The percentiles with a reported wake-up times (week prior to completing the questionnaire) at or below each time are plotted versus clock hours after midnight. The percentiles are plotted for 6 groups: a) absolutely certain DSPD 1, b) fairly certain DSPD 2, c) questionable DSPD 3, d) unlikely or very doubtful DSPD 4–5, e) the DSPD-C, and f) a representative sample of San Diego adults.

As shown in Fig. 3, 4, the bedtimes and uptimes reported for the prior week by DSPD groups overlapped DSPD-C somewhat more than did the HO scores or actigraphic sleep acrophases. Nevertheless, as was the case for HO scores and sleep acrophases, reported bedtimes and uptimes still differed very significantly between DSPD and DSPD-C groups (p < 0.001), and the variances were significantly greater in the DSPD group (p < 0.001). Bedtime and uptime distributions of the DSPD-C group and the representative sample were almost superimposable. However, small percentages of the representative sample had bedtimes earlier or later than the range for the DSPD-C, reflecting the exclusion of suspected cases of ASPD or DSPD and shiftworkers from among the DSPD-C group but not from the representative sample. Among DSPD and DSPD-C, the Spearman Rank Order Correlation of reported bedtimes with age was not significant.

### Correlations of measures

Among DSPD participants and the representative sample, combined, the sleep acrophase was strongly correlated with the wrist activity acrophase (r = 0.83, P < 0.001) and somewhat less associated with the wrist illumination acrophase (r = 0.44, P < 0.001). The objective sleep acrophases correlated r = 0.80 (P < 0.001) with reported bedtimes for the week before the questionnaire was completed and r = 0.83 (P < 0.001) with reported uptimes.

Only the DSPD group both had wrist activity recordings and had completed the MES scales. It was surprising that within the DSPD participant group (definite, probable, or questionable), correlations of the Horne-Östberg scale with the observed sleep acrophase and the reported bedtimes and uptimes were not significant, and the HO correlation with the activity acrophase was quite weak (r = -0.16, P < 0.05). The correlation of the HO scale with the reported average time of going to sleep since age 21 was somewhat greater (r = -0.31, P < 0.001). The correlation of the HO and BALM MES scales within the restricted range of the DSPD group was only r = 0.68 (P < 0.001).

However, expanding the range analyzed by including the DSPD-C participants with the DSPD group, the correlation of the HO and BALM scales was r = 0.96 (P < 0.001). Over this expanded range, the correlation of the HO scale with reported bedtimes was r = -0.72 (P < 0.001), with reported uptimes r = -0.66 (P < 0.001), and with usual sleep time (since age 21) r = -0.74 (P < 0.001). The BALM was slightly less strongly correlated with bedtimes, uptimes, and usual sleep times.

### Discriminating DSPD from DSPD-C

Contrasting the DSPD group (certain, probable, or questionable) with the DSPD-C, there were one DSPD and one DSPD-C participant with scores of 41 on the HO MES. There was 1 DSPD-C out of N = 202 with score < 41, and there were 2 DSPD (1–3) out of N = 183 with scores > 41. In the BALM, there were 5 DSPD with scores of 28 and 5 scoring > 28, whereas there were 5 DSPD-C with BALM of 28 and 5 < 28. Thus, the HO scale separated the groups somewhat better than the BALM.

With the HO scale alone, binary logistic regression correctly classified 180 of 183 DSPD cases (absolutely certain, fairly certain, or questionable) and 200 of 201 DSPD-C (certain). Adding the BALM as an independent variable correctly classified one additional DSPD, but the BALM by itself misclassified 14 participants. Reported bedtime in the prior week by itself misclassified 36 participants. The combination of HO, BALM, reported bedtime, and age correctly classified all DSPD and DSPD-C.

### Sleep symptoms

Based on sleep and health questionnaires, in one-way contrasts, DSPD-C tended to report their sleep as "moderately" or "very" satisfactory, whereas DSPD were more likely to report sleep as "somewhat troubled" or "troubled" (P < 0.001). DSPD-C were more likely to report getting "just enough sleep" (P < 0.001). DSPD were more likely to report that their sleep was "abnormal" (P < 0.001). DSPD were 10 times as likely to report "insomnia" and 6 times more likely to have consulted a doctor for help with a sleep problem (P < 0.0001). DSPD reported trouble falling asleep 2.9 (SD 2.4) times per week, whereas controls reported such trouble 0.7 times per week (SD 1.3) (P < 0.001). DSPD-C reported a mean sleep latency of 15 min (SD 12) and DSPD 37 min (SD 42) (P < 0.001). DSPD reported a past-week total sleep time of 7.2 (SD 1.5) hours and DSPD-C reported 7.4 (SD 1.1) hours (P < 0.05). Likewise, DSPD reported 30 (SD 50) min of being awake during the night, whereas DSPD-C reported 18 (SD 38) min (P < 0.01), yet there was no significant difference in reported trouble staying asleep. DSPD also reported snoring slightly more commonly and awakening earlier than intended more commonly (P = 0.005). DSPD reported trouble waking up 5 nights per week, whereas DSPD-C reported an average of 1 night a week (P < 0.001). Reported bedtimes of DSPD for the prior week averaged 2:00 AM whereas those of controls averaged 22:45 (P < 0.001, see Fig. 3). Likewise, reported uptimes averaged 10:00 for DSPD and 06:47 for controls (P < 0.001, see Fig 4). Similarly, DSPD reported trouble waking up in the morning 4.8 (SD 2.5) times per week, while DSPD-C reported this problem only 1.0 (SD1.7) times per week (P < 0.001). DSPD reported very slightly more snoring (P < 0.005) and awakening gasping for breath than controls (P = 0.02), but were very slightly less likely to report being treated for sleep apnea (P = 0.01). There was no significant difference in reported leg twitching or kicking during sleep. In terms of reported sleep quality, DSPD had a mean PSQI of 7.3 (SD 3.8) indicating poor sleep quality, whereas DSPD-C had a mean PSQI of 3.8 (SD 2.8) (P < 0.001, Mann-Whitney U test). Moreover, of the DSPD-C, 88% had a PSQI lower than the median of 7 for DSPD and 17% of DSPD had a PSQI equal or lower to the DSPD-C control median of 3.

### Mood

About 44% of DSPD but only 21% of DSPD-C reported having seen a counsellor for treatment of emotional problems (P < 0.001), and likewise, 51% of DSPD vs. 23% of controls reported that there had "been a time when you were depressed or down most of the day nearly every day for as long as two weeks," a common screening question for major depression (P < 0.001.) The mean scores were 6.0 for DSPD and 3.4 for controls on the QIDS-SR depression scale (P < 0.001). Similarly, 4% of DSPD vs 2% of controls reported having been hospitalized for depression (NS). Also, 38% of DSPD vs. 26% of controls reported "grandparents, parents, brothers, sisters, or children ever had definite depression" (P < 0.02). Only about 1% of each group reported having been treated for mania or bipolar disorder (NS), and only about 10% of each group reported a family history of bipolar disorder (NS). Similarly, the mean score on the Mood Disorder Questionnaire was 3.6 for DSPD and 3.0 for controls (NS). Using this Mood Disorder Questionnaire, 9 DSPD and 6 controls met screening criteria for a past history of mania (NS). The SPAQ global seasonality score was not significantly associated with DSPD (mean 5.47 SD 5.03 and mean 4.70 SD 3.99 for DSPD and DSPD-C respectively, P > 0.10). No controls but 8% of DSPD reported having been treated for a circadian rhythm disorder or with light treatment (P < 0.001). DSPD reported having taken hypnotics 2.7% of their lives, whereas controls reported only 0.2% on average (P < 0.001). Reporting no lifetime use of hypnotics were 81% of DSPD and 96% of controls. Likewise, DSPD reported taking stimulants (amphetamines, methylphenidate, or modafinil) 2% of their lives vs. 0.3% for controls (P < 0.02). Similarly, DSPD reported taking antidepressants 5.9% (SD 13.7%) of their lives vs. 1.5% (SD 6.4%) for controls (P < 0.001).

### Developmental history

In childhood, 50% of DSPD reported that they went to sleep "much later" than other children their age and 32% reported "somewhat later," whereas the percentages for controls were 3% and 19% (P < 0.001, Mann-Whitney U). Similarly, in adulthood, 72% of DSPD reported they went to sleep "much later" than other people their age and 25% "somewhat later," whereas the corresponding percentages for controls were 4% and 20% (P < 0.001, Mann-Whitney U).

### Height and BMI

DSPD and DSPD-C did not differ significantly in height. For BMI, both gender (P = 0.025) and case/control (DSPD vs DSPD-C, P = 0.002) were significant factors in ANCOVA with age as a significant covariate (P = 0.001), but there was no significant gender/diagnosis interaction. The BMI of DSPD averaged 33.0, and the BMI of DSPD-C averaged 30.5.

### Eye color and ancestry

Participants had self-described eye color, which might influence the synchronizing efficiency of light exposures. Among the DSPD and DSPD-C combined, 29% were blue-eyed, 9% grey or green eyed, 15% hazel eyed, and 47% brown-eyed. The DSPD and DSPD-C groups did not differ significantly by eye color, either considering the distribution of 4 color categories, or considering them by order of iris lightness. The groups were also generally similar in aspects of reported ancestry.

### General health

In the past month, antacids were used by 15% of DSPD and only 5% of DSPD-C (Chi-Square = 11.31, P = 0.001). Antidepressants were used by 23.8% of DSPD and 10.4% of DSPD-C (Chi-Square = 12.39, P < 0.001). Hypnotics were used by 4.9% of DSPD and 0.5% of DSPD-C (Chi-Square = 7.33, P = 0.007). Melatonin was used by 7% of DSPD and 1% of DSPD-C (Chi-Square-9.45, P = 0.002). Over-the-counter hypnotics were used by 3.2% of DSPD and 0.5% of DSPD-C (Chi-Square = 4.1, P < 0.05). Use of anticonvulsants, antihypertensives, bronchodilators, calcium channel blockers, cardiac medications, corticosteroids, cytotoxic drugs, diuretics, hormones (largely birth control), insulin, laxatives, major tranquilizers, minor tranquilizers, narcotics, and miscellaneous other medications did not differ significantly between DSPD and DSPD-C. However, DSPD took an average of 0.84 of this latter group of medications, whereas DSPD-C took an average of 0.64 (t = 2.05, two-tailed, P < 0.05).

### Familiality

When asked about biological relatives with a tendency to go to bed late and get up late, DSPD reported that at least one grandparent was characteristically late 16.5%, very late 10.2%, or extremely late 3.9%, whereas DSPD-C reported 7.5%, 5.5%, and 0.7% respectively (P = 0.001, Mann-Whitney U). Similarly, DSPD reported fathers were 20.8% late, 12.3% very late, and 8.4% extremely late, whereas DSPD-C reported 22.3%, 6.3%, and 1.1% respectively (P = 0.005, Mann-Whitney U). Further, DSPD reported mothers were 21.8% late, 13.3% very late, and 12.7% extremely late, whereas DSPD-C reported 23.8%, 4.9%, and 2.2% respectively (P < 0.001, Mann-Whitney U). The lateness of fathers and mothers did not differ significantly overall. However, DSPD female probands reported that fathers were later than male probands reported them (P = 0.02, Mann-Whitney U), but the mothers were described with similar lateness by male and female DSPD. DSPD reported that they had at least one brother or sister who was very late (22.7%) or extremely late (13.3%), whereas DSPD-C reported 14.7% and 7.9% respectively (P = 0.005, Mann-Whitney U). At least one child was reported very late or extremely late by 34.4% of DSPD but only 20.3% of controls (P < 0.001, Mann-Whitney U).

## Discussion

These data characterize a case series of DSPD volunteers and compare them to normal controls (both DSPD-C and a previously-collected representative sample). The DSPD-C were well-matched by age, gender, and ancestry. The sleep timing of DSPD-C was demonstrably normal in the sense that their reported bedtimes and uptimes were almost superimposable on the distributions of a representative sample of San Diego adults. DSPD cases went to bed and arose about 3 hours later than DSPD-C on average, but the DSPD group had a considerably wider distribution of sleep times, reflecting a proportion who retired and arose very late indeed. Subjectively reported bedtimes and uptimes and MES scores for DSPD-C and activity acrophases and the acrophases of sleep inferred from wrist activity for the representative sample were reasonably consistent in indicating the degree of delay experienced by the DSPD cases. It must be recognized, however, that the Horne-Östberg (HO) MES scores, bedtimes, and sleep acrophases for cases and controls in San Diego might occupy somewhat different numerical ranges from those in other communities, where average bedtimes might be later.

There was almost no overlap of HO MES scores between those classified as definite probable or possible DSPD cases and those classified as DSPD-C. The two groups were distinguished 98% by the HO criterion of 41. Likewise, there was little overlap in BALM scores, but there was somewhat more overlap in self-reported bedtimes and uptimes. The superiority of the HO scale over bedtimes for distinguished DSPD may be somewhat tautological, resulting from the way in which recruitment and case-classification were achieved. Defining a DSPD case group primarily by the HO scale, we could examine how bedtimes and other characteristics compared in DSPD and DSPD-C. That the HO scale distinguished somewhat better than the BALM may reflect the investigators' classification methods, but since the HO was slightly better correlated with bedtime, uptime, and actigraphic measures than the BALM, it did appear that in these samples, the HO distinguished cases somewhat more effectively. Ultimately, comparison with genetic findings may best reflect the relative validity of these two MES scales and determine whether they serve classification of circadian disorders better than indicators of bedtime or uptime. It is interesting that in a case series based on clinical contacts, greater overlap was observed for bedtimes and physiologic circadian indicators between ASPD and normal groups and between normals and DSPD [58]. Perhaps genetic findings will also reflect whether MES scales or clinical misalignment criteria better identify heritable circadian abnormalities. In addition, genetic data may clarify whether the 3% of the population reporting both trouble falling asleep and trouble waking up suffer a milder masked form of DSPD, with a misalignment of sleep time and sleep propensity, even when their bedtimes may be within the normal range [5].

Although these results supported many previous studies which have observed that older adults tend to go to bed and arise somewhat earlier than young adults, the effect size was very small. It did not appear that adjustment for age would be important in discriminating DSPD from normal people.

Over the broad range of DSPD cases and DSPD-C, the HO and BALM scales were well correlated with bedtimes, uptimes, and sleep acrophases, but within the group of DSPD cases, these correlations were poor. This suggests that though the HO and BALM scales distinguished well between cases and controls, MES scales may be an unsatisfactory indicator of the severity of DSPD within the DSPD group. Perhaps bedtimes and especially uptimes would be the best indicator of the extent to which DSPD may be handicapping.

**Figure 5:**
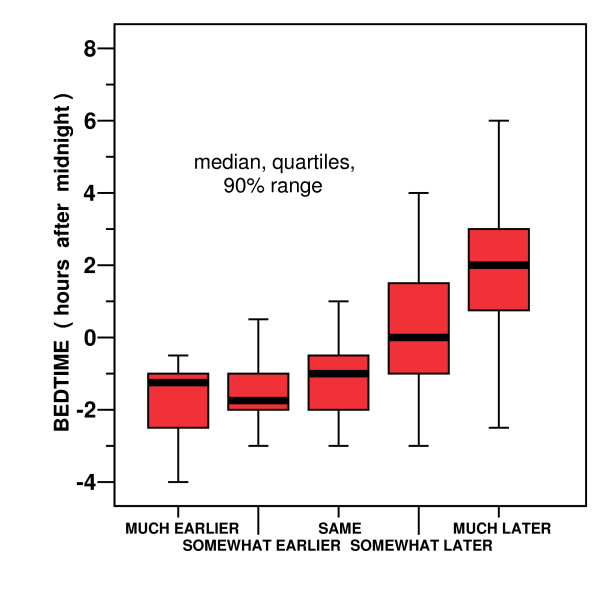
**Self reported comparison of bedtimes with other people**. Self-reported bedtimes (in clock time after midnight) are reported for participants who stated that on average (after age 21) they went to sleep much earlier, somewhat earlier, about the same time, somewhat later, or much later that other people their age. Thick horizontal lines are medians. Red boxes show the 2^nd ^and 3^rd ^quartiles. Bars show the upper and lower 5%.

Recently, there has been interest in MES scales which ask participants to compare their morning-evening preferences to those of "most people" [59-61]. Our question asking people to compare their bedtimes with most people their age produced responses with considerable overlap in bedtimes (Fig. 5). This would lead us to be sceptical that subjective comparisons to other people would be an optimal approach to discerning morningness-eveningness traits. People who perceived their own bedtimes as earlier or later than most people did not always retire correspondingly early or late in reference to our representative sample. Even within a single complex society, differences in peer groups related to income, occupation, and social background may produce marked differences in perceived normal bedtimes and in desired bedtimes. It remains to be established whether misalignment between objective sleep times and desired sleep times, perhaps indicative of genetic polymorphisms, may best be recognized by reference to a person's individual desired sleep times, to the perceived bedtimes of their peers, or to the bedtimes of the overall surrounding community.

There was considerable overlap between case and control groups in measures of distress about sleep. Thus, 17% of DSPD had a PSQI score as low as the control median, whereas 12% of DSPD-C had a PSQI at least equal to the DSPD median. Some DSPD cases were able to arrange their sleep schedules well enough to avoid distressed sleep, whereas others suffered persistent trouble falling asleep and trouble arising in the morning. It appears that those suffering DSPD who must force their sleep schedule into a biologically adverse time will habitually experience lying in bed having trouble falling asleep. As a consequence, they may develop fear of the bed and anxiety about retiring. Even when able to retire at a biologically appropriate time, this negative conditioning may lead to psychophysiologic insomnia and sleep disturbance.

As a group, we found that the DSPD participants had very irregular sleep patterns. They commonly had prolonged midsleep awakenings. Many appeared to nap frequently at all times of day. By comparing the actigraphic records with the sleep logs, we gained the impression that some DSPD participants were drowsing while watching television late at night, a considerable time before they went to bed and turned out the lights. Unfortunately, some actigraphic records were so interspersed with immobile intervals that we regarded our minute-by-minute actigraphic scoring of sleep as quite unreliable, though only in rare cases did we lack confidence in the overall estimate of the circadian pattern. Despite the degree of sleep disturbance – or perhaps because of the napping out of bed – it appeared that DSPD cases got about an hour more actigraphic sleep each 24 hours than our representative sample. However, because the representative sample were older on average, because different actigraphs and scoring algorithms were used in the two samples, and because of numerous other details of the sleep measurements, we would not consider this distinction in actigraphic sleep times between DSPD cases and the representative sample to be reliable. Note that DSPD-C subjectively reported slightly more sleep than DSPD in the week prior to completing their questionnaires.

DSPD seemed to experience less illumination per 24 hours than had the representative sample, but the effect size was modest. Because the photometric measurement devices used in the two studies were quite different in design and performance, we do not know if this difference was reliable. It would be logical that since DSPD arise late and are up later at night than the normal population, they would be out of bed less in bright daylight. Less daylight exposure, in turn, could promote susceptibility to DSPD, since daylight usually functions to advance circadian rhythms, particularly soon after a person awakens. Increasing a patient's exposure to bright light soon after awakening is an accepted treatment approach for DSPD [62].

Depression comorbid with DSPD was noted in the pioneering description of DSPD [63] and has been described by several observers [64-69]. Our data confirm that DSPD cases had higher current depression ratings and a greater life-time history of unipolar depression, depression treatment, and anti-depressant use than DSPD-C. DSPD cases also had a greater family history of depression, supporting the possibility that DSPD and depression share genetic susceptibility factors. Nevertheless, there may be behaviorally-mediated mechanisms for comorbidity between DSPD and depression. For example, the lateness of DSPD cases and their unusual hours may lead to social opprobrium and rejection, which might be depressing. Moreover, late awakenings and the rush to school or work might lead to reduced morning daylight exposure, which may predispose to depression. Conversely, the withdrawal and fatigue associated with depression, by reducing outdoor activities and daylight exposure, could lead to circadian phase delay. Perhaps genetic studies will clarify these associations. We attempted to carefully identify any association of bipolar disorder (history of mania) with DSPD, but we found no evidence of significant association with bipolar disorder.

Insofar as medications indicate health concerns, DSPD took more medications for sleep and mood, more medications in general, and more antacids. Perhaps the circadian misalignment produced by DSPD tends to cause epigastric discomfort, as is often reported by shift workers, which might lead to increased use of antacids and increased snacking. On the other hand, DSPD may have averaged relatively high BMI because of lack of exercise, resulting from their schedules.

## Conclusion

Finally, these studies tend to confirm that a tendency to being a nightowl is partly familial and presumably caused in part by genetic susceptibilities. Family members of DSPD cases were far more likely to be described as very late in their habits than family members of DSPD-C, and family members also had more depression. We do not think the preponderance of women in our case series indicates that DSPD is more common among women: indeed, a variety of evidence suggests the contrary. Rather, we would attribute the preponderance of women to their greater willingness to participate in such the studies, since a similar preponderance was found in the control groups. One finding of possible interest was that DSPD female probands reported that fathers had later bedtimes and uptimes than male probands reported. This might be explained if DSPD is transmitted in part through an X chromosome (and therefore, not from fathers to sons), possibly as a recessive trait, but the evidence was not robust enough to be persuasive. None of the initially-recognized circadian system genes are found on the X Chromosome, but FMR1 is one X Chromosome gene which might be a candidate for polymorphisms related to DSPD [70].

In future reports, we hope to extensively examine associations of DSPD cases with polymorphisms in several of the circadian system genes.

## Competing interests

The authors declare that they have no competing interests.

## Authors' contributions

DFK helped design the study, supervised the recruitment and clinical classification of participants, and participated in data analyses and writing. KMR helped plan the study, recruited most of the cases, and participated in data entry, analyses, and writing. SAI helped design the study, assisted in recruiting, and participated in manuscript drafting. CMN helped design the study and participated in manuscript preparation. WK helped design the study, supervised DNA assays, and participated in manuscript review. JRK helped design the study, supervised DNA preparation, and participated in manuscript review. All authors read and approved the final manuscript.
